# Childhood Obesity: Immune Response and Nutritional Approaches

**DOI:** 10.3389/fimmu.2015.00076

**Published:** 2015-02-24

**Authors:** Thea Magrone, Emilio Jirillo

**Affiliations:** ^1^Department of Basic Medical Sciences, Neuroscience and Sensory Organs, University of Bari, Bari, Italy

**Keywords:** atherosclerosis, children, diabetes, immunity, microbiota, nutrition, obesity, oxidative stress

## Abstract

Childhood obesity is characterized by a low-grade inflammation status depending on the multicellular release of cytokines, adipokines, and reactive oxygen species. In particular, the imbalance between anti-inflammatory T regulatory cells and inflammatory T helper 17 cells seems to sustain such a phlogistic condition. Alterations of gut microbiota since childhood also contribute to the maintenance of inflammation. Therefore, besides preventive measures and caloric restrictions, dietary intake of natural products endowed with anti-oxidant and anti-inflammatory activities may represent a valid interventional approach for preventing and/or attenuating the pathological consequences of obesity. In this regard, the use of prebiotics, probiotics, polyphenols, polyunsaturated fatty acids, vitamins, and melatonin in human clinical trials will be described.

## Introduction

Nowadays, childhood obesity has become an epidemic all over the world. In terms of estimates, 200 million school-age children are overweight/obese and 40–50 million of them are obese (1).

According to current literature, 70% of obese adolescents become obese in adult life also in relation to parental obesity (2–4). Of note, overweight and obesity seem to be irrespective of the socio-economic conditions and income status, as reported by Lobstein et al. (5), Popkin and Gordon-Larsen (6), and Wang and Lobstein (7). However, in developed countries, social inequality represents a risk of developing obesity (8). On the other hand, genetic, epigenetic, environmental factors, and inappropriate life styles (imbalanced diets and sedentary life) greatly contribute to the development of obesity and related diseases, such as insulin resistance, type 2 diabetes, and metabolic syndrome (MetS) (9).

With regard to the etiology of childhood obesity (10), the prenatal period should be considered as a critical period for its development. Excessive or low calorie intake by the mother during the prenatal and perinatal period seems to predispose to obesity in postnatal life stages (10). Children born from diabetic mothers or mothers who smoked during pregnancy are more exposed to the risk of obesity and type 2 diabetes (11). Also, breastfeeding seems to reduce the risk of overweight/obesity. In addition, the so-called “obesity rebound” at 5–7 years old seems to be very critical for the development of obesity and risk of obesity in adulthood (10). Puberty is another critical period in life especially in females, in terms of obesity risk since early menarche (<11 years) in females predisposes to obesity in a more remarkable way than later menarche (>14 years) (10). In these females, early menarche leads to obesity in adolescence and in adulthood, while in 70% of obese adolescent males weight tends to normalize at later ages in comparison to 20% of obese adolescent females.

From a genetic point of view, humans have acquired the ability to deposit fats and utilize them under a condition of starvation. However, this characteristic turned into a disadvantage in western societies where the abundant consumption of food leads to an increase of fat deposits. As far as nutritional factors are concerned (12), evidence has been provided that obese children consume more fat than non-obese children, thus leading to higher body mass index (BMI). In addition, fats tend to deregulate control mechanisms for body weight thanks to a greater amount of energy *per gram* they provide. Fats are more palatable with a smaller satiating effect in comparison to proteins and sugars and the actual amount ingested. Another nutritional aspect in obese children and adolescent is the irregular eating pattern with a lower frequency of breakfast consumption.

Physical activity (PA) represents another important factor in the development of obesity since reduced PA correlates with excess body weight (12). Sedentary life due to playing video-games and watching television affects basal expenditure. In particular, watching television also increases calorie intake for consumption of foods which, in turn, are publicized by the same programs children are watching. Finally, an association has been found between short sleep and obesity in the sense that sleep duration is inversely associated to BMI and waist circumference.

The excess of fat negatively influences health and/or wellbeing in children. Data reported by Freedman and associates (13) demonstrated that childhood obesity is associated to the risk of cardiovascular events in adult life. In addition, Lauer and associates (14) described some factors affecting the relationship between childhood and adult cholesterol levels with special reference to early development of atherosclerosis in children. Metabolic (high levels of triacylglycerols, low levels of high density lipoprotein-cholesterol) and clinical modifications (high blood pressure and defective glucose metabolism) are part of the MetS, which represents one of the major complications of childhood obesity (15). For instance, type 2 diabetes and cardiovascular disease represent sequelae of MetS.

On these grounds, the aim of the present review is to describe the profile of the immune response in obese children, taking into consideration that obesity is an “inflammatory” disease. Furthermore, interventional studies aimed at preventing obesity and/or attenuating the immune-inflammatory profile will be illustrated.

## Immunity in Childhood Obesity

Obesity is associated to a condition of systemic inflammation, on the one hand (16), and to an impaired immunity, on the other hand (17). For instance, obese individuals were more susceptible to the H1N1 influenza during the 2009 pandemia (18). In obese individuals, visceral adipose tissue (VAT) is the major source of pro-inflammatory cytokines, which, in turn, lead to insulin resistance (16, 19). Experimentally, obese animals produce higher amounts of adipose tissue-derived tumor necrosis factor (TNF)-α in comparison to the lean counterpart (20), while in obese humans Pradhan and associates (21) documented the association between C-reactive protein, interleukin (IL)-6, and the risk of developing type 2 diabetes. Quite interestingly, macrophages with an inflammatory phenotype have been detected in the context of VAT of obese people (22, 23). They account for 40–60% of VAT immune cells in obesity and differ from the phenotype of macrophages contained in lean adipose tissue (22, 23). Obesity-associated macrophages in VAT are M1 “classically activated macrophages.” They secrete in large amounts TNF-α, IL-6, IL-12, IL-1β, and monocyte chemotactic protein-1 (MCP-1), as well as nitric oxide (NO) (24). On the contrary, in lean VAT M2 macrophages are “alternatively activated macrophages,” which secrete IL-10, IL-1 receptor antagonist, and arginase 1, thus exerting anti-inflammatory activities. The contingent of obesity VAT macrophages seems to be in part residential or, in alternative, migrates into VAT from remote sites under the effect of MCP-1. M1 macrophages seem to favor insulin resistance and in murine models their deactivation protects against insulin resistance (25, 26). Of note, VAT macrophages and adipocytes share common functions in terms of pattern of cytokine released and insulin resistance induction (27). It is worthwhile mentioning that, in obese mice, neutrophils and mast cells also play an inflammatory role, while the number of eosinophils is decreased (28). The above described cellular profiles promote insulin resistance in murine obesity (28).

As far as adaptive immune responses are concerned in obese mice, VAT contains higher numbers of T helper (Th) CD4+, T cytotoxic CD8+ cells, and B cells. In particular, Th1 cells produce interferon (IFN)-γ *in vitro* (29). IFN-γ, in turn, polarizes M1 macrophages, also increasing release of TNF-α.

Conversely, obese mice lacking IFN-γ expression or T-cell receptor β-deficient mice are more protected with regard to inflammatory cell infiltration of VAT (29, 30). T regulatory (Treg) cells are decreased in both obese mice and humans (31, 32). In murine VAT, depletion of Treg cells led to increased insulin levels and reduced insulin receptor signaling (32). On the other hand, expansion of Treg cell contingent in high-fat-diet-fed mice and increased secretion of IL-10 led to a significant reduction of blood glucose levels, insulin resistance, and glucose tolerance. These data suggest the anti-inflammatory role exerted by Treg cells *via* release of IL-10, which suppresses obesity-induced inflammation. Han and associates (33) have reported that insulin bears receptors on Treg cells, thus decreasing IL-10 release by activating AKT/mammalian target of rapamycin signaling pathway. These results suggest that high insulin levels in obesity play an inflammatory role by impairing Treg cell-induced suppression.

Th17 cells are increased in obese mice and in humans, thus leading to enhanced expression and release of IL-17 (34–36). However, evidence has been provided that γδ T cells and neutrophils can also produce IL-17 and, therefore, Th and Th17 cells are not the only source of this cytokine (37, 38). According to Zúñiga (37), these various sources of IL-17 may explain some contradictory results obtained in IL-17 knockout mice which are overweight and become obese as results of a high-fat diet compared to controls. Despite these controversial results in mice, there is evidence that obesity is associated to autoimmune diseases in both mice and humans likely *via* a Th17-dependent mechanism (39). Finally, as recently reported by Erbel and associates (40), IL-17A plays a pathogenetic role in advanced murine and human atherosclerosis, which is a complication of the obese status.

CD8+ cells accumulate into VAT in murine obesity and their depletion led to reduction of macrophage infiltration and amelioration of insulin sensitivity (41, 42). Adoptive transfer of CD8+ cells into CD8-deficient mice increased inflammatory cytokine production in the context of VAT. Jiang and associates (43) have documented that CD8+ cells in VAT are activated by IFN-γ released by Th1 cells and, moreover, they express high levels of the integrin CD11a, which promotes infiltration of CD8+ cells into VAT.

B-cell infiltration into VAT seems to precede T cell and macrophage homing into this tissue. According to Winer and associates (44), B cells into VAT provoke insulin resistance, modulating T cells and producing immunoglobulin (Ig)G, which account for insulin resistance. Conversely, obese B null mice lack inflammatory cytokines, produce high levels of IL-10, and are protected against insulin resistance.

Major features of immune alterations in obese people are indicated in Table 1.

**Table 1:** **Alterations of innate and adaptive immunity in human obesity**.

VAT tissue produces pro-inflammatory cytokines, which are responsible for insulin resistance (16, 19)
The association between C-reactive protein, IL-6, and the risk of developing type 2 diabetes has been documented (21)
M1 macrophages with an inflammatory phenotype have been found in obese people VAT (22, 23)
T regulatory cells are decreased in human obesity (31, 32)
Th17 cells are increased in obese humans (34–36)
CD8+ cells express high levels of the integrin CD11a, which promotes their infiltration of CD8+ into VAT (43)
B cells into VAT provoke insulin resistance, modulating T cells and producing immunoglobulin gG, which account for insulin resistance (44)

## The IL-10/IL-17 Ratio and Type of Diet

In recent years, it has become clear that some lifestyle factors, including dietary habits, alcohol consumption, exercise, and smoking play an important role in the control of both post-prandial lipemia and inflammation. Advancing data suggest that dietary anti-oxidants may influence both metabolic and inflammatory markers linked to low-grade systemic inflammation (45, 46). In general terms, low-grade inflammation seems to be determined by the ratio between IL-17 and IL-10. IL-17 has been associated to the pathogenesis of multiple autoimmune diseases, such as rheumatoid arthritis, multiple sclerosis, and inflammatory bowel diseases (47). IL-17 also plays a crucial role in host defense upon bacterial and fungal infections by recruiting neutrophils and producing antimicrobial peptides (AMPs) (48). Th17 cells release multiple molecules of IL-17 (A–F) (49) and IL-17F was also found to be co-expressed in Th17 cells, thus contributing to host defenses, inflammatory, allergic, and autoimmune functions of Th17 cells (50).

Interleukin-10, a product of Treg cells (51) inhibits cytokine production, particularly IFN-γ by T and natural killer (NK) cells, and proliferation of T cells, performing primarily at the level of antigen-presenting cells (52). IL-10 also inhibits other monocyte/macrophage functions, including oxidative burst, NO, pro-inflammatory cytokine production, and cytotoxicity (53).

The relationship between dietary habits and IL-10/IL-17 ratio in children has been stressed out in a recent paper (Vitale et al., submitted). Schoolchildren with normal weight received healthy eating recommendations and, then, BMI values, PA, and levels of salivary IL-17 and IL-10, respectively, were followed-up at enrollment, after 6 months and after 1 year (Vitale et al., submitted). Results of this follow-up demonstrated that increase in BMI and reduced PA was associated to a decrease in IL-10 and an increase in IL-17 salivary levels in one group of children. In the other group characterized by reduction in BMI and increase in PA, IL-10 salivary levels were higher than those detected in the case of IL-17. Variations in BMI were oscillating within normal ranges.

With special reference to healthy food recommendations provided to children involved in this trial (Vitale et al., submitted), they are in agreement with those elaborated by the American Heart Association for children aged 2 years and older (54). The suggested diet relies on fruits and vegetables, whole grains, low-fat and non-fat dairy products, beans, fish, and lean meat. These general recommendations associated to other recent dietary guidelines (55, 56) are primarily based on low intakes of saturated and trans fat, cholesterol, added sugar and salt, energy intake, and PA according to the Mediterranean diet (MedDiet) model (57, 58). In this framework, Knoops and associates (59) have shown that in European men and women aged 70–90, adherence to a Mediterranean-style diet was associated to a lower rate of all-cause mortality. Taken together, the combination was associated to a mortality rate of about one-third of those with none or only one of these protective factors. In a large prospective survey involving about 22,000 Greek adults, adherence to a Mediterranean-style diet and death was associated to approximately 2/9 increment in the MedDiet score with a 25% reduction in total mortality (60).

There is experimental evidence that a combination of diet and exercise reduces adipose tissue derived-inflammation and macrophage involvement (61, 62).

In the above mentioned study (Vitale et al., submitted), increased levels of IL-17 in children with higher BMI seem to be responsible for a low-grade inflammation attributable to the intake of hypercaloric food [junk food; see also Ref. (59)] and reduced PA. Instead, in children with lower BMI, elevated IL-10 levels support an anti-inflammatory status, likely dependent on the strict adherence to healthy dietary recommendations and PA. In this framework, it is worthwhile mentioning that post-prandial stress is associated to a condition of low-grade inflammation (63) with a massive increase of free radicals and pro-inflammatory cytokines (64). In the long run, inflammatory mediators might cause damage of intestinal barrier function, leading to endotoxin leakage into the portal blood (32). Increased endotoxin levels, in turn, might provoke weight gain, insulin resistance, and a higher degree of inflammation, a condition referred to as “metabolic endotoxemia,” also associated to cardiovascular disease (65).

The major achievements related to the relationship between dietary habits and IL-10/IL-17 ratio in normal weight children are summarized in Table 2.

**Table 2:** **Type of diet and IL-10/IL-17 ratio**.

Normal weight children who attended dietary recommendations and practiced PA exhibited a reduction of BMI and an increase in IL-10 salivary levels and a decrease in IL-17 salivary levels (Vitale et al., submitted)
Normal weight children who did not attend dietary recommendations and did not practice PA exhibited an increase in BMI and in IL-17 salivary levels while IL-10 salivary levels were decreased (Vitale et al., submitted)

## Oxidative Stress in Obesity

Obesity is associated to excessive production of reactive oxygen species (ROS) from different sources such as mitochondrial respiratory chain and nicotinamide adenine dinucleotide phosphate (NADPH) oxidase (66–69). This event leads to the development of insulin resistance and MetS, thus deregulating adipokine and pro-inflammatory cytokine release.

In obese mice with insulin resistance, an adipocyte fatty acid-binding protein has been identified, which is modified by 4-hydroxynonenal, a lipid peroxidation-derived aldehyde (70). Thus, adipose proteins, which play a role in cellular stress, lipotoxicity, or insulin signaling are carbonylated as a result of the oxidative process. Furthermore, in obese mice programed by early weaning, a series of metabolic disturbances were detected such as visceral adiposity, hypertension, dyslipidemia, hepatic steatosis, and high concentrations of hepatic triglycerides (70). These alterations were associated to plasmatic and hepatic oxidative stress supported by elevated amounts of thiobarbituric acid-reactive substances (markers of lipid peroxidation) and decreased activities of superoxide dismutase (SOD) and glutathione peroxidase. This experimental model is an example of the link between obesity and oxidative stress. In another study, evidence has been provided that malondialdehyde (another biomarker of lipid peroxidation), carbonylated proteins, and SOD activity were increased in testicular tissue and serum of obese rats (71).

In humans, studies by Keaney and associates (72) have clearly demonstrated the association between oxidative stress and obesity by monitoring urinary isoprostanes, which are a reliable index of oxidative stress *in vivo* (73). Of note, weight loss due to dietary changes and increased PA was able to significantly reduce urinary levels of the isoprostane 8-epi-prostaglandin F2-α, thus supporting the link between obesity and oxidative stress (74).

From a pathogenic point of view, evidence has been provided that hydrogen peroxide-induced oxidative stress leads to the differentiation of pre-adipocytes into adipocytes *via* transcription factors such as CCAAT/enhancer-binding protein-β and peroxisome proliferator-activated receptors-γ (75). Another molecule, protein kinase C (PKC), is involved in the adipocyte differentiation (76). In fact, PKC deficiency increases fatty acid oxidation and reduces fat storage or its loss protects obese mice against hepatic steatosis and insulin resistance (77, 78).

On the other hand, obesity can induce oxidative stress in adipocytes *via* production of ROS by mitochondria which can be increased in response to a high-fat diet (79, 80). In this framework, it has been reported that mitoNEET present in the outer mitochondrial membrane causes increased lipid storage, thus augmenting the mass of murine adipose tissue and, ultimately, leading to obesity (81). Quite interestingly, NOX-2 (the catalytic core of NADPH oxidase) has been found to be over-expressed in hypercholesterolemic and obese children (82). In these children, the enhanced NOX-2-dependent oxidative stress and reduction of flow-mediated arterial dilation indicated a condition of endothelial dysfunction. In addition, the increased oxidative stress in obese people decreases the production of adiponectin, an adipokine which inhibits plasminogen activator inhibitor (PAI)-1, IL-6, and TNF-α (83). Adipocyte-induced increase of TNF-α and PAI-1 is responsible for a prothrombogenic status and insulin resistance in obese people (20, 84). Finally, inhibition of adiponectin production increases insulin resistance and promotes atherosclerosis (85, 86).

According to Furukawa and associates (83), adipocytes produce ROS and the resulting oxidative stress is able to induce insulin resistance in skeletal muscle and adipose tissue and decreased secretion of insulin by pancreatic β cells, thus, generating atherosclerosis and hypertension. Furthermore, oxidative stress and hyperglycemia generate advanced glycation end products (AGEs). They bind to cellular receptors [advanced glycation end products receptors (RAGEs)], thus contributing to the condition of low-grade inflammation in obesity (87). In addition, a deficit of soluble RAGEs in obesity has been associated to low levels of adiponectin and increased oxidative stress (88).

The effects of oxidative stress in human obesity are outlined in Table 3.

**Table 3:** **Oxidative stress and human obesity**.

Oxidative stress decreases the release of adiponectin with an increase in TNF-α and PAI-1, thus leading to a prothrombogenic status and insulin resistance (83)
In obese children, the overexpression of NOX-2 and dependent oxidative stress suggests a condition of endothelial dysfunction (82)
Adipocytes produce ROS, thus leading to decreased secretion of insulin by pancreatic β cells which is associated to atherosclerosis and hypertension (83)
Generation of AGEs in response to oxidative stress and hyperglycemia contributes to low-grade inflammation and low levels of adiponectin in the presence of a deficit of soluble RAGEs (87, 88)

On these grounds, in a recent report (89), salivary NO concentration was determined in overweight/obese children, and, then, compared to that of normal weight and underweight counterparts. In children, eating was assessed by considering the youth healthy eating index (YHEI) (90) and compared to BMI, activity/inactivity, and salivary NO concentration. Data documented that in overweight/obese individuals, BMI positively correlated to YHEI scores, inactivity, and NO concentration, respectively, while BMI inversely correlated to PA. Previous studies have demonstrated the role of PA in the prevention of obesity (91, 92). In addition, in the HELENA project (93), it has been demonstrated that more physically active and leaner children undergo higher energy intake than that observed in less active adolescents with more fat-mass. The progressive increase in body weight results from a daily cumulative effect of even a small caloric excess, which may be related to the reduction of PA (94). Data previously discussed (89) as well as other epidemiological studies confirm the inverse relationship between PA and BMI (95, 96). The findings related to the increase in salivary NO concentration in overweight/obese children (89) are in accordance with a series of reports (97–100) in which various other biomarkers were monitored for assessing the inflammatory profile in obesity (101). Particularly, NO participates to inflammation as a product of M1 macrophages, whose activities are depending on TNF-α, IL-1β, and IL-6 release (102–104). In this direction, detection of salivary NO in overweight/obese children may be interpreted as a biomarker of inflammation. However, other studies have found no modifications of NO levels in obese children (105).

## Immune Profile in Childhood Asthma and Obesity

Evidence has been provided that a correlation exists between obesity and asthma in children (106, 107), which is often independent from allergic sensitization, in accordance with same observation in adults (108).

Asthma incidence in children may be ascribed to air pollution, environmental tobacco smoke, infectious agents, and detrimental dietary habits (109).

Adipokines such as leptin, adiponectin, resistin, and visfatin have been object of interest in childhood obesity and asthma clinical manifestations. Leptin is a plasma protein involved in the regulation of food intake (110) and is able to exert pro-inflammatory effects on dendritic cells (DCs), NK cells, T cells, B cells, and Treg cells (111). Adiponectin exerts anti-inflammatory activities, inhibiting IL-6 and TNF-α production (112). Resistin is expressed in human macrophages, bone marrow, spleen, and peripheral lymphomonocytes and at low levels in adipose tissue (113). It is able to activate NF-κB, thus promoting a pro-inflammatory cytokine release. Visfatin, known as a pre-B-cell colony-enhancing factor, is able to exert insulin-mimetic effect (114, 115). In addition, visfatin has been shown to induce chemotaxis of phagocytes as well as production of IL-1, TNF, IL-6 (116).

On these bases, Magrone and associates (117) have investigated the immunological mechanisms involved in asthma and obesity in terms of cytokine and adipokine release. Eighty children were enrolled and were divided into four groups: asthmatic obese, obese, asthmatic, and control children. For each group, BMI was calculated and asthma was defined following the Global Initiative for Asthma (GINA) Guidelines (118). IL-2 and IFN-γ serum levels were higher in asthmatic obese children than those of controls. IL-4 serum levels were undetectable, while IL-13 serum levels were not statically significant between groups. On the other hand, IL-1β, IL-6, and IL-8 serum levels were increased in asthmatic obese and obese children in comparison to asthmatic children and controls.

With regard to adipokines, leptin serum levels were higher in obese and asthmatic obese in comparison to asthmatic children and controls. Conversely, a significant reduction of serum adiponectin levels particularly in asthmatic obese children was detected. In conclusion, one can hypothesize that increased levels of leptin may account for the enhancement of Th1 responses (119), while adiponectin reduction may be responsible for a diminished release of IL-10 by Treg cells (120), thus worsening the pro-inflammatory status.

**Figure 1:**
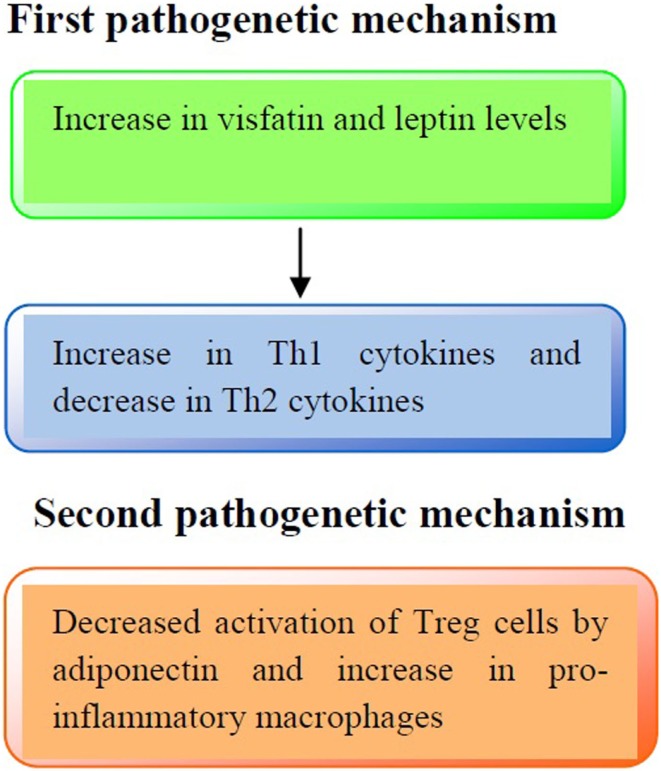
**Pathogenetic mechanisms involved in the immune profile in asthmatic obese children**. First pathogenetic mechanisms: increased production of visfatin and leptin serum levels may lead to an increased release of Th1 cytokines (IL-2, IFN-γ) and a decreased release of Th2 cytokines (IL-4, IL-13), respectively. Second pathogenetic mechanism: adiponectin reduces the activation of Treg cells and increases pro-inflammatory cytokines production (IL-1β, IL-6, and IL-8).

The immune profile in obese asthmatic children is illustrated in Figure 1.

## Role of Intestinal Microbiota in Obesity

Evidence has been provided that intestinal microbiota composition is altered in obese people, even including children, as recently reviewed by Kabat and associates (121). Accordingly, intestinal microbiota is active on both innate and adaptive immunity. With regard to innate immunity, commensal bacterial products [e.g., lipopolysaccharides (LPS)] bind to Toll-like receptors (TLRs) and Nod-like receptors present on gut immune and non-immune cells. LPS, then, binds to TLR-4 on epithelial cells, soliciting release of the AMP ASReg III-γ. This AMP is also produced by lamina propria (LP) DCs *via* TLR-5 stimulation, which in turn, secrete IL-23, an inducer of IL-22 producing Th17-type cells. The role of IL-22, in this context is to amplify the release of other AMPs in the gut (121). Furthermore, digestion of plant polysaccharides by microbiota gives rise to short chain fatty acids (SCFAs), which, in turn, induce release of IL-18 from intestinal epithelial cells *via* binding to the G-protein coupled receptor (GPCR). Among SCFAs, acetate seems to protect epithelial barrier function, mediating an anti-apoptotic response. Taken together, all these gut microbiota-elicited activities seem to be protective to the host.

With special reference to the adaptive immunity, microbiota actively participates to intestinal IgA production *via* release of B-cell activating factor, a proliferation-inducing ligand and transforming growth factor (TGF)-β by intestinal epithelial cells and DCs, thus leading to differentiation of B cells into IgA-producing plasma cells. Also, follicular DCs promote differentiation of IgA-producing plasma cells, as main producers of TGF-β in the Peyer’s patches (PP). Moreover, interaction between Th17-like cells and DCs favors IgA production within PP. Th17-like cells also promote T-cell homing to the LP *via* soluble form of LTa3 lymphotoxin, thus affecting T helper follicular (ThF) cell function. Also ATP, generated by certain commensals, contributes to the induction of Th17 cells which, in turn, differentiate into ThF cells, thus promoting IgA production within PP. Polysaccharide A (PSA) from *Bacteroides fragilis*, SCFAs, and TGF-β induced by Clostridia (IV, XIVa, XVII) promote Treg cell differentiation in the colon. PSA acts on TLR-2 exposed on DCs, while SCFA operate *via* GPCR43 signaling (121). From these data, it is evident that microbiota-induced IgA production and Treg cell differentiation in the gut confers protection under healthy circumstances.

The altered microbiota in obesity subverts the protective mechanisms above illustrated. For instance, increase in segmented filamentous bacteria induce release of serum amyloid A by epithelial cells which acting upon DCs leads to differentiation and induction of Th17 cells endowed with inflammatory activities (122, 123). Furthermore, there is evidence that modifications of microbiota composition in early life may increase the risk to developing obesity in later life. For instance, children delivered by caesarian section exhibit higher risk to become obese in adolescence when compared to children born by vaginal delivery (124). Furthermore, breast-milk-fed children have less risk to become obese than infant formula-fed children (125). Also, exposure of antibiotics in early life may alter the composition of microbiota as observed in mice which underwent changes in hepatic lipid and cholesterol metabolism, thus leading to adiposity (126). In humans, a correlation has been found between early-life antibiotic use and obesity (127) and other studies have documented that presence/absence of specific microbiota components can modulate immune response (128).

## Interventional Studies in Childhood Obesity

Prevention seems to be the most appropriate strategy to combating obesity epidemic. According to a number of reports, there are some major factors to be considered in the prevention of obesity development (129–132). For instance, maternal factors encompass monitoring of weight before conception and during pregnancy. Breastfeeding may represent a favorable factor in terms of reduced risk of obesity.

Dietary factors are based on limited consumption of sugar-sweetened beverages and meals with servings of vegetable and fruits, avoiding fast foods, and encouraging limited portions of food.

Physical activity is based on levels of activity from moderate to vigorous for one or more hours/day. Sedentary activity (television, play station) should be limited to less than 2 h/day after age two. Settings where food and PA can be influenced are represented by schools and preschool institutions as well as after-school care services. Built environment encompasses walking and cycling networks, parks, and recreation facilities. Home environment should also be studied as a possible factor of obesity prevention but the limitation is represented by the heterogeneity of homes and possibility of access.

## Prebiotics

By definition, prebiotics are non-digestible dietary fibers, which are able to stimulate both growth and activity of gut bacteria. The anti-obesogenic effects of prebiotics have mostly been evaluated in experimental studies. In prebiotic-fed genetically obese mice reduction of circulating endotoxins, pro-inflammatory cytokines and intestinal permeability was reported (133). In rats with steatohepatitis induced by a high-fat diet, lactulose treatment reduced liver inflammation and endotoxemia (134). Human clinical trials based on the effects of prebiotics on obesity development are very scanty. In infants receiving formula enriched in prebiotics (galacto-oligosaccharides and fructo-oligosaccharides at 9:1 ratio), an increase in Bifidobacteria was reported, thus suggesting the possibility to influence adipocyte growth *via* modulation of microbiota composition (135). In patients with non-alcoholic steatohepatitis (NASH), administration of oligofructose reduced serum aminotransferases and insulin levels (136). Of note, in western countries NASH is very common in obese children (137).

## Probiotics

Various strains of bacteria have been found in the gut of obese and lean humans and, according to recent data, it seems that just smaller modifications of intestinal commensals may account for weight gain (138). On the other hand, in overweight adolescents, the response to diet and PA was dependent on the microbiota composition present before treatment (139). In this direction, gut bacteria such as *Ruminococcus bromii* and *Eubacterium rectale* were prevalent in individuals under a diet rich in resistant starch who responded to a dietary weight loss program (140). Furthermore, *Lactobacillus (L.) gasseri* SBT2055 (LG2055) administration to overweight subjects could lead to a significant reduction of abdominal adiposity (141).

Just recently, fecal microbiota transplantation has been applied to patients with inflammatory bowel disease and obese patients (142). In particular, transfer of intestinal microbiota from lean donors to obese recipients attenuated clinical manifestations of MetS (143).

With special reference to pediatric obesity, the ratio between *Bacteroidetes* and *Firmicutes* seems to play a role in weight gain (144). Also, the size of Enterobacteriaceae, such as *Desulfovibrio* and *Akkermansia* (*A*.) *muciniphila* were found to be related to pediatric obesity (145). These last findings are also supported by experiments in obese mice which underwent reduction of fat-mass gain, endotoxemia, adipocyte-induced inflammation, and insulin resistance following treatment with *A. muciniphila* (146).

Other clinical trials in pregnant mothers have documented that administration of *L. rhamnosus* 4 weeks before expected delivery up to 6 months after delivery could limit excessive weight gain during the first 2 years of life but not between 2 and 4 years (147). However, maternal supplementation with probiotics in the first trimester of pregnancy did not modify prenatal and postnatal growth rates (148, 149). In this framework, evidence has been provided that milk from obese mothers is enriched in *Staphylococcus* and *Lactobacillus* with lower counts of *Bifidobacterium* when compared to that of normal weight women over the first 6 months of breastfeeding (150). The role of milk microbiota on the development of neonatal microbiota needs to be further investigated.

Synbiotics are a mixture of pro- and prebiotics which, when ingested, are able to modulate gut microbiota and intestinal immunity (151). In a recent clinical trial, Kelishadi and associates (152) have administered overweight children with a synbiotic (Protexin-London, England) composed by a combination of viable Lactobacilli of human origin and fructo-oligosaccharides, as prebiotics. Treated subjects exhibited a significant reduction in weight as well as in TNF-α and IL-6 with an increase in adiponectin in comparison to the placebo group. However, the modifications of inflammatory markers were dependent on weight reduction.

## Fatty Acids

Fatty acids exert important biological functions in the body as a substrate for energy and the formation of membranes, also acting as regulators of genetic expression (153). Excessive consumption of saturated fatty acids or an altered ratio between omega-3 polyunsaturated fatty acids (n-3 PUFAs) and n-6 PUFAs leads to obesity, diabetes, neurodegenerative disease, and cancer (154, 155). n-3 PUFAs and their derivatives, eicosapentaenoic acid (EPA) and docosahexaenoic acid (DHA), are able to reduce plasma triglyceride levels and body weight (156, 157). Furthermore, n-6 PUFAs promote excessive adipose tissue growth, while n-3 PUFAs inhibit adipogenesis, while promoting storage and accumulation of mature adipocytes (158–162). In terms of early interventions at the level of maternal inflammation, studies in transgenic Fat-1 mice have documented that increase in n-3/n-6 PUFA ratio diminished fetal-placental lipid exposure, thus limiting adverse metabolic effects in adult offsprings (162). This therapeutic model may be applied for preventive therapy in obese pregnant women. However, as stated in a recent review by Hauner and associates (163), results on the prevention of childhood obesity obtained through modification of fatty composition during pregnancy and lactation are still contradictory and inconsistent.

In a very recent paper (164), it has been reported that consumption of n-3 PUFAs in obese adolescents along with dietary restriction improved anthropometric parameters, while decreasing plasma triglyceride levels. These effects correlated to a reduced hypoxia in subcutaneous adipose tissue (164).

## Polyphenols

Polyphenols encompass flavonoids and non-flavonoids (resveratrol) compounds, which are widely distributed in the vegetal kingdom. They are mostly contained in fruits, vegetables, and cereals, and, therefore, contained in large amounts in MedDiet (165).

Polyphenols are endowed with anti-oxidant and anti-inflammatory activities and moderate consumption of red wine has been shown to prevent cardiovascular disease according to the French paradox (166–168). Experimentally, administration of resveratrol from red grapes to obese rats reduced visceral obesity and triglycerides and low-density lipoprotein plasma concentration, thus decreasing the risk of hypertension, dyslipidemia, and steatosis (169). Furthermore, flavonoids hampered both transcription factors and differentiation of pre-adipocytes into mature adipocytes (170). The *in vitro* demonstration that polyphenols from fermented grape marc differentiate and activate peripheral human Treg cells further supports the anti-inflammatory role of these natural compounds (171). Just recently, evidence has been provided that cocoa power supplementation ameliorated the pro-inflammatory profile in high fat-fed obese mice (172). Same results have been obtained with the administration of epigallocatechin-3-gallate (EGCG) in high fat-fed mice (173). In obese women administration of green tea (EGCG) did not affect body weight, fat-mass, energy, homeostasis, cardiometabolic risk factor, and liver function (174). Also, results by Li and associates (175) demonstrated that green tea supplementation did not influence blood pressure among overweight and obese adults. Conversely, in normal weight obese syndrome subjects, regular consumption of dark chocolate was useful in maintaining a good atherogenic profile for its effects on HDL cholesterol, lipoprotein ratios, and inflammatory markers (176).

In general terms, consumption of natural oxidants contained in polyphenols with the diet may afford protection against cardiovascular disease acting upon lipid profile, endothelial function, and inflammatory mediators (177, 178).

## Melatonin

Melatonin is a pineal hormone endowed with anti-oxidant properties, thus, preventing nitro-oxidative stress mediated by peroxynitrites (179). Melatonin also exhibits anti-inflammatory activities inhibiting ciclooxygenase-2 and inducible NO synthase and acting upon transcriptional pathway involved in inflammation, such as NF-κB, AP-1, Nrf2, as well as PI3K/Akt and MAPK kinase (180, 181). In view of its beneficial activities, melatonin has successfully been used in rats with MetS diminishing insulin resistance, release of TNF-α and IL-6 from adipocytes, low-density lipoprotein, and very low-density lipoprotein plasma levels and body weight (182). Evidence has been provided that melatonin can promote weight loss in rodents *via* browning of white adipose tissue (183) and this may represent a new approach to treat human obesity (184). In fact, melatonin is a non-toxic compound widely distributed in foodstuffs, such as olive oil, wine, coffee, tea, walnuts, and grapes. Experimentally, a combination of resveratrol and melatonin afforded protection in a model of myocardial infarction (185). In this context, over the past few years, some clinical trials have documented the beneficial effects of melatonin in patients with MetS in terms of amelioration of blood pressure, lipid pattern, and oxidative stress markers (186–188).

## Vitamin D

Vitamin D exerts anti-inflammatory activities, acting on DCs, which in turn, induce activation of Treg cells. This vitamin possesses specific receptors, so-called Vitamin D receptors (VDR), on gut epithelial and immune cells, while bacterial colonization seems to affect distribution and expression of VDR (189). In humans, vitamin D deficiency has been associated to asthma and increased BMI (190–192). Furthermore, it has been hypothesized that gut microbiota and vitamin D may be linked cofactors in the pathogenesis of childhood asthma and obesity (193).

On these grounds, vitamin D may represent another possible target of interventional studies in asthma-obesity but birth cohort studies based on maternal and neonatal diet, gut microbiome, immune response, and vitamin D-mediated immune regulation are needed for asthma/obesity prevention.

The major nutritional attempts to prevent/attenuate childhood obesity are illustrated in Table 4.

**Table 4:** **Some effects of natural products on obese humans**.

Prebiotics, non-digestible dietary fibers, which are able to stimulate both growth and activity of gut bacteria (194), induce increase in Bifidobacteria in infants (135) and decrease in serum aminotransferases and insulin levels in NASH (136)
Probiotics, live bacteria, which when administered in adequate amounts confer a health benefit to the host (195). Administration of *L. gasseri* to overweight subjects reduced abdominal adiposity (141). Fecal microbiota transplantation attenuated clinical manifestation of MetS (143). *L. rhamnosus* administration to pregnant mothers limited excessive weight gain during the first 2 years of life (147). Synbiotic administration to overweight children reduced weight gain and pro-inflammatory cytokine release (152)
n-3 Polyunsaturated fatty acids consumption in obese adolescents along with dietary restriction improved anthropometric parameters, while decreasing plasma triglyceride levels. These effects correlated to a reduced hypoxia in subcutaneous adipose tissue (164)
Polyphenols (flavonoids and non-flavonoids compounds) present in fruits vegetable and cereals exert anti-inflammatory and anti-oxidant activities (165)
Melatonin, a pineal hormone, has been shown to be protective in patients with MetS in terms of improvement of blood pressure, lipid profile, and oxidative biomarkers (186–188)
Vitamin D deficiency has been associated to asthma and increased BMI and, therefore, together with gut microbiota alterations may lead to childhood asthma and obesity outcome (193)

## Conclusion

Nowadays, obesity is an epidemic in western and westernized societies, thus representing one of the major consequences of food-related disease (196). Besides appropriate dietary habits and PA, an anti-inflammatory profile in response to food antigens should be maintained throughout life span. For instance, post-prandial low-grade inflammation is normally compensated by dietary components, e.g., polyphenols, which activate gut Treg cells (197).

A continuous intake of junk food since childhood may account for the outcome of a systemic inflammation in overweight/obese adults.

In interventional studies, another important aspect is represented by the identification of gut microbial components involved in the development of obesity. In view of the diversity of human intestinal microbiota (198), its variations among different obese individuals should be investigated also in terms of interpersonal microbiome differences (199). For instance, in the case of treatment with probiotics generating a smaller effect size, the personal microbiome effect which is very large may mask the more feeble effects of treatment. Therefore, methods for the study of microbiome should be borrowed from the scientific areas and adjusted for analyzing massive data as in the case of obese people.

In conclusion, more appropriate diets (e.g., MedDiet) or supplements containing natural products (polyphenols, n-3 PUFA, vitamins, synbiotics) are highly recommended to prevent or attenuate the noxious effects of obesity. In this last regard, in a very recent review, Casas and associates (200) have stressed out the immune protective effect of MedDiet, which may act on various immune biomarkers, such as molecules involved in the stability of atheromatous plaque.

## Conflict of Interest Statement

The authors declare that the research was conducted in the absence of any commercial or financial relationships that could be construed as a potential conflict of interest.

## Abbreviations

AGEs, advanced glycation end products; AMP, antimicrobial peptide; BMI, body mass index; DCs, dendritic cells; DHA, docosahexaenoic acid; EGCG, epigallocatechin-3-gallate; EPA, eicosapentaenoic acid; GPCR, G-protein coupled receptor; IFN, interferon; Ig, immunoglobulin; IL, interleukin; LP, lamina propria; LPS, lipopolysaccharides; MCP, monocyte chemotactic protein; MedDiet, Mediterranean diet; MetS, metabolic syndrome; n-3 PUFAs, omega-3 polyunsaturated fatty acids; n-6 PUFAs, omega-6 polyunsaturated fatty acids; NADPH, nicotinamide adenine dinucleotide phosphate; NASH, non-alcoholic steatohepatitis; NK, natural killer; NO, nitric oxide; PA, physical activity; PAI, plasminogen activator inhibitor; PKC, protein kinase C; PP, Peyer’s patches; PSA, polysaccharide A; RAGEs, advanced glycation end products receptors; ROS, reactive oxygen species; SCFAs, short chain fatty acids; SOD, superoxide dismutase; TGF, transforming growth factor; Th, T helper; ThF, T helper follicular; TLRs, toll-like receptors; TNF, tumor necrosis factor; Treg, T regulatory; VAT, visceral adipose tissue; VDR, vitamin D receptor; YHEI, youth healthy eating index.
